# Language-Guided Segmentation of Medical Images: A Review of Foundation Models

**DOI:** 10.3390/bioengineering13070803

**Published:** 2026-07-13

**Authors:** Saqib Qamar

**Affiliations:** 1 Department of Intelligent Systems, KTH Royal Institute of Technology, 10044 Stockholm, Sweden; sqama@kth.se; 2 Faculty of Computing and Information Technology (FCIT), Sohar University, Sohar 311, Oman

**Keywords:** vision-language models, medical image segmentation, foundation models, Segment Anything Model, text-prompted segmentation

## Abstract

Vision-language foundation models have transformed medical image segmentation over the past three years. These models pair large image encoders with text prompts, so a single model can segment many anatomical structures, lesion types, and imaging modalities through natural language. This survey reviews vision-language foundation models designed for medical image segmentation. We describe the technical background from contrastive vision-language pretraining to the Segment Anything Model and its medical variants. We propose a three-part taxonomy that covers text-prompt-guided models, large-language-model-embedded architectures, and hybrid frameworks. We examine adaptation strategies such as full fine-tuning, Low-Rank Adaptation, adapters, and prompt engineering. We organize the literature by modality and cover computed tomography, magnetic resonance imaging, pathology, chest radiography, and ultrasound. We discuss clinical uses such as organ segmentation, tumor delineation, and radiotherapy planning. We summarize evaluation metrics and benchmark datasets. We identify four open challenges: prompt dependence, mask hallucination, slow volumetric inference, and limited annotated data. We close with a research roadmap for trustworthy deployment, multimodal pretraining, and clinical integration.

## 1. Introduction

Medical image segmentation provides the pixel-level basis for many clinical workflows, supporting diagnosis, treatment planning, surgical guidance, and quantitative disease monitoring. Convolutional encoder-decoder networks such as nnU-Net long served as the standard segmentation tool across imaging modalities [1,2], and vision transformers later added longer-range context [3,4]. Self-adaptive Mamba-like attention mechanisms extend this design space by combining state-space dynamics with transformer-style cross-attention [5]. These architectures improved accuracy on many benchmarks but still required task-specific training data [6,7]. The shift to foundation models, trained once on very large datasets and then adapted to many downstream tasks [8], has changed how segmentation systems are built. In computer vision, the Segment Anything Model showed that a single model can handle many object types with simple visual prompts [9], while large language models brought general-purpose text understanding [10,11]. Vision-language models bridge these two lines: Contrastive Language-Image Pretraining (CLIP) established the recipe of dual encoders trained with a contrastive loss on hundreds of millions of image-text pairs [12,13], which was later ported to medical imaging through MedCLIP, BiomedCLIP, GLoRIA, and BioViL [14,15,16,17,18]. Text prompts opened a new way to control segmentation: instead of bounding boxes or click points, a clinician can write a phrase such as “liver tumor in arterial phase CT” and obtain a mask. Early work in this text-prompted paradigm includes LViT and the CLIP-Driven Universal Model [19,20]; more recent systems such as BiomedParse and BiomedParse-V scale it to dozens of modalities and hundreds of object types [21,22], and others couple large language models with segmentation decoders to reason over complex queries [23,24,25]. The pace of work has accelerated, with hundreds of papers published in 2024 and 2025 alone [26,27,28]. This rapid growth motivates a focused survey. Earlier reviews cover foundation models in medicine broadly [26,28], report generation [27], transformers [7], or CLIP in medical imaging [29], and a 2026 review surveys medical image segmentation foundation models [30]; Table 1 compares these with our work. The narrower question of vision-language foundation models for medical segmentation, and its text-driven workflows rather than SAM’s visual prompts, has not yet received in-depth treatment. This survey fills that gap.

This survey makes three contributions. First, to our knowledge we provide the first taxonomy organized specifically around how language guides medical image segmentation. Second, we map the architectural and training choices of more than fifty recent methods to their reported benchmark performance. Third, we identify open problems and solutions around trustworthiness, efficiency, and clinical integration, with close attention to 2025–2026 work, which is about thirty percent of the references here. The motivation is substantial: manual segmentation of one CT scan can take thirty minutes to several hours, and radiotherapy contouring one to four hours per patient [36,37,38]. By automating a first-pass contour, foundation models can substantially reduce this manual effort, and in reported studies of automated organ-at-risk contouring have cut interaction time severalfold [36,37,38]; potential further benefits such as improved cross-institution consistency and centralized updates are plausible but not yet established at scale [8,39,40]. Natural-language interfaces also lower the barrier to quantitative analysis [23,24,41], and open models such as MedSAM, BiomedCLIP, BiomedParse, and LLaVA-Med are central to this effort [15,21,42,43,44].

To keep the scope explicit: this review centres on language-guided (text-driven) medical image segmentation. Inside the scope are text-prompt-guided models, large-language-model-embedded architectures, and hybrid vision-language segmentation frameworks. General vision-language models, the Segment Anything Model and its purely visual-prompt variants, radiology report-generation models, and non-text-prompted deep-learning segmentation networks are outside the primary scope and are included only as background or as baselines for comparison, where they are grouped separately and labelled as context.

**Figure 1:**
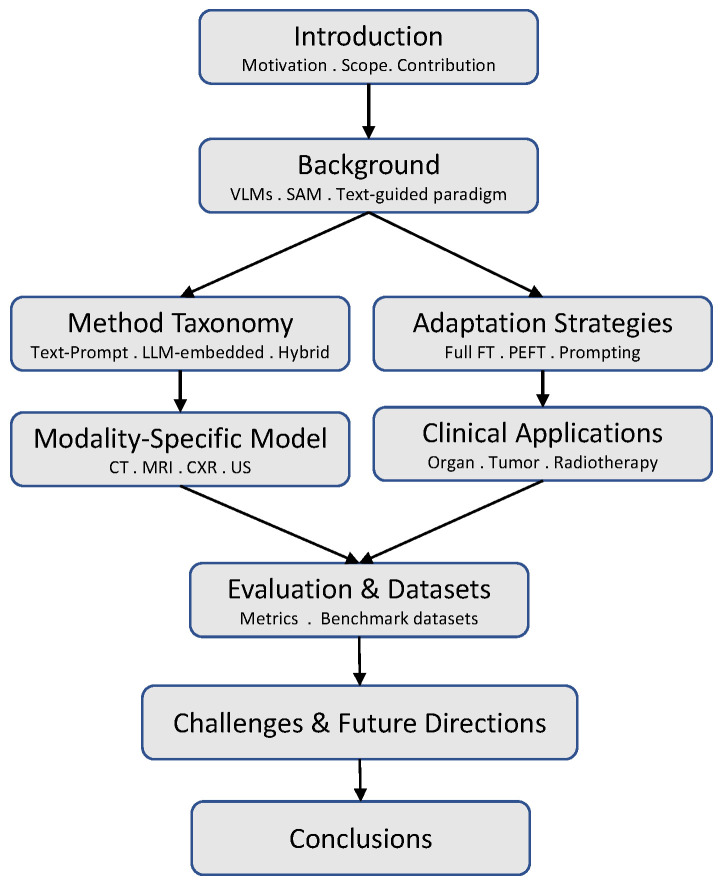
Organization of this survey, showing the major sections and the relationships among background, methods, adaptation, modalities, applications, evaluation, and the combined challenges and future directions.

We organize the survey as follows in Figure 1. Section 2 describes the review methodology. Section 3 gives technical background on vision-language models, the Segment Anything Model and its medical variants, and the text-guided paradigm. Section 4 presents a three-part taxonomy of text-prompt-guided, LLM-embedded, and hybrid models. Section 5 covers adaptation strategies (full fine-tuning, parameter-efficient methods, and prompt engineering) [45,46]. Section 6 and Section 7 review models by imaging modality and by clinical application. Section 8 summarizes evaluation metrics and datasets [47,48,49,50]. Section 9 discusses challenges and future directions, and Section 10 concludes.

## 2. Review Methodology

This is a scoping review rather than a systematic review, so it does not report pooled statistics; instead it maps the design space of vision-language foundation models for medical image segmentation. To make the literature selection transparent and reproducible, we followed the process below.

**Sources, search terms, and date range.** We searched Google Scholar, PubMed, arXiv, and the proceedings of CVPR, ICCV, MICCAI, NeurIPS, and ICLR, and we tracked the reference lists of the models we included up to January 2026. We combined vision-language terms (“vision-language model”, “CLIP”, “foundation model”, “large language model”) with segmentation terms (“segmentation”, “Segment Anything”, “SAM”) and medical terms (“medical”, “clinical”, “radiology”, “CT”, “MRI”, “pathology”, “chest X-ray”, “ultrasound”), together with method names such as “text-prompted”, “referring segmentation”, and “reasoning segmentation”. We focused on work published between 2021 (the release of CLIP) and January 2026, with emphasis on 2024–2026 methods; foundational earlier work is cited for context.

**Inclusion and exclusion criteria.** We included methods that (i) perform medical image segmentation and (ii) use language or a vision-language component. We also included a limited set of SAM-based and non-text-prompted deep-learning models as baselines and background, and radiology report-generation models where they share components with segmentation systems; these are labelled as context in Table 2. We excluded classification-only or retrieval-only vision-language models with no segmentation output, purely visual-prompt methods with no language component, and works without an accessible description of the method.

**Screening, selection, and data extraction.** Records identified through the searches were screened by title and abstract and then by full text against the criteria above; after removing duplicates and out-of-scope records, we retained the methods discussed in the text and the fifty-three methods compared in Table 2. For each retained method we recorded year, architecture, target modality, training and evaluation datasets, adaptation strategy, peak reported Dice where available, peer-review status, and code availability. We recorded whether each method is peer-reviewed or a preprint.

## 3. Background

**Figure 2:**
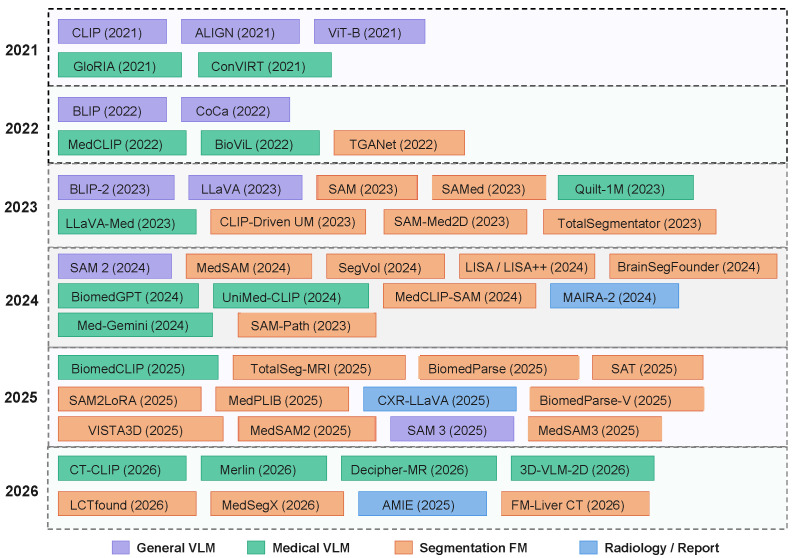
Timeline of key vision-language and segmentation foundation models from 2021 to 2026. General vision-language models (purple) laid the architectural foundations. Medical vision-language models (teal) adapted these to clinical data. Segmentation foundation models (coral) combined prompt-based interaction with medical domain knowledge. Radiology and report generation models (blue) extended these to clinical workflows. Years reflect first public release or peer-reviewed publication.

This section reviews the building blocks of vision-language foundation models for medical image segmentation. We start with general vision-language models, then describe the Segment Anything Model and its derivatives, and finally explain the text-guided segmentation paradigm that links these two lines of work. Figure 2 places the key models on a timeline from 2021 to 2026.

### 3.1. Vision-Language Models

Vision-language models learn aligned image-text representations through contrastive learning on image-caption pairs [12,13]. CLIP introduced this recipe at scale: separate image and text encoders map inputs into a shared space where a contrastive loss aligns matched pairs, enabling zero-shot classification [12]. Later designs explored tighter modality fusion and caption bootstrapping to clean web data, and instruction tuning produced assistants that answer free-form questions about images, the basis for later medical multimodal models.

Medical imaging needs specialized models because visual and textual distributions differ from web data [14,29]: images focus on anatomy and pathology, and text comes from reports with technical vocabulary [51,52]. MedCLIP decoupled image-text pairs to overcome the small size of paired medical data [14], GLoRIA introduced global-local contrastive learning to align text tokens with image regions [16], and BioViL/BioViL-T refined the approach for chest radiology, with BioViL-T modeling temporal change across follow-up scans [17,18]. BiomedCLIP extended the recipe to fifteen million biomedical image-text pairs from PubMed Central, covering radiology, pathology, and microscopy [15], and PMC-CLIP collected a related large-scale dataset [53]. LLaVA-Med adapted instruction tuning to biomedical figures and captions [42], and large language models were specialized for clinical question answering. RadFM and a later generalist radiology model targeted three-dimensional volumes [54,55]. Med-Gemini reached expert-level performance on several clinical benchmarks [44,56,57], clinician-VLM collaboration improved radiology report generation [58], and CT-CLIP brought contrastive pretraining to volumetric chest CT paired with structured reports [59]. Pathology has its own line of vision-language foundation models. PLIP trained on histopathology image-caption pairs from medical Twitter [60], and Quilt-1M assembled a larger image-text dataset from educational videos [61]. UNI and Virchow scaled image-only foundation models for whole-slide images [62,63], and H-optimus-0 is a recent open-source example. PathChat and PathAsst add conversational interfaces [64], while RetCCL and masked-image-modeling backbones support retrieval and self-supervised pretraining [65,66]. These models supply rich visual representations that can be coupled with segmentation heads.

Across these models, paired text supervision yields image features that transfer well to dense tasks such as segmentation, and many recent segmentation methods build on pretrained CLIP-style or self-supervised encoders rather than training from scratch [67,68]. A practical limitation for segmentation is that standard CLIP produces a single global feature with limited spatial structure [12]; CLIP Surgery and related designs expose spatial features for dense prediction [69]. Adapting natural-image models to medicine is essential because vocabulary and semantics differ substantially from web data [14,29]. Domain-specific text encoders such as Bio_ClinicalBERT, BioBERT, and PubMedBERT represent medical text far better than generic CLIP encoders [14,51,52], and GatorTron specializes LLMs for electronic health records [70]. Multilingual support for non-English documentation remains an open need [56,71].

### 3.2. Segment Anything and Medical Variants

The Segment Anything Model (SAM) was a turning point for general-purpose segmentation [9]. Trained on over one billion masks across eleven million images, it accepts visual prompts (points, boxes, rough masks) and returns a mask via a heavy Vision Transformer image encoder [3], a lightweight prompt encoder, and a small mask decoder, enabling near-real-time inference on cached image features. SAM’s SA-1B data was generated by a model-in-the-loop process in which expert annotators refined model-suggested masks, an approach later reused to augment partially annotated medical data [9,43,72,73]. SAM transfers well to natural images, and 2023 zero-shot evaluations on medical data [31,32,33,74,75] found a consistent pattern: it works well on high-contrast objects with clear boundaries but struggles on small lesions, low-contrast structures, and 3D volumes, with chest radiography and dermatology outperforming abdominal CT and histopathology. The encoder also lacks medical-specific features and processes each slice independently, which motivated medical SAM variants [43,72,73,76]. MedSAM is the most influential of these efforts [43,77]. It collected more than 1.5 million image-mask pairs across ten modalities and fine-tuned the SAM mask decoder while keeping the image encoder mostly frozen, using bounding-box prompts. It achieves strong Dice across radiology, pathology, and microscopy, and MedSAM2 extends to images and short videos [77]. SAM-Med2D scaled the dataset to roughly 4.6 million images and 19.7 million masks and added encoder adapter layers to bridge the natural-medical gap [72,78]. SAM-Med3D rebuilt the architecture for volumetric inputs with 3D positional encodings and attention, trained on more than twenty thousand 3D images [73]. SegVol supports more than two hundred categories with spatial and text prompts [79], and SegFM3D continues the 3D foundation-model line [80]. Several variants target specific needs: SAMUS adapts SAM to noisy ultrasound [81], SegmentAnyBone to multi-sequence bone MRI [82], AdaptiveSAM adds bias-tuning and text prompts for surgical scenes [83], and 3DSAM-adapter extends 2D SAM to promptable volumetric tumor segmentation [84], building on early task-specific adaptation [85]. Large experimental and empirical studies characterized how best to adapt SAM for medical use [31,34]. SAM 2 (2024) supports images and videos through a memory mechanism with an improved encoder and temporal reasoning; MedSAM2 extends it to medical images and short videos [77]. SAM 3 adds concept-aware segmentation by incorporating semantic knowledge into the prompt framework [86], MedSAM3 brings concept-aware prompting and parameter-efficient fine-tuning to the third generation [87], and SAM2LoRA reaches state-of-the-art retinal fundus segmentation while updating fewer than five percent of the parameters [88].

These models share a common limitation: they are built around visual prompts, so a clinician must still place points or boxes for each object of interest. This is workable for individual cases but does not scale to large studies or to natural-language-driven applications, the gap that the text-guided segmentation paradigm addresses.

### 3.3. Text-Guided Segmentation Paradigm

Text-guided segmentation produces a mask from an image and a natural-language description of the target, ranging from a class label such as “liver” to a clinical phrase such as “hyperdense lesion in the right lobe” or a free-form question. The model must translate the text into a spatial decision, a task at the intersection of vision-language alignment and dense prediction.

Three lines of work converged to make text-guided segmentation possible. Open-vocabulary segmentation in natural images uses CLIP text embeddings as classifiers for arbitrary concepts, with CLIPSeg and CRIS adapting the idea to dense prediction through small decoders and mask transformers [12,89,90]. Referring-expression segmentation, in models such as LAVT, ReSTR, and CRIS trained on RefCOCO-style data, segments objects described by short phrases by fusing visual and textual features early in a transformer. Reasoning segmentation then added world knowledge: LISA emits a special segmentation token whose hidden state decodes into a mask [23], LISA++ extended it with instance-level reasoning and multi-turn dialog [24], subsequent studies generalized the paradigm to video and 3D data [25], and SEEM unified multiple prompt types in a single mask decoder [91]. Text-guided segmentation in medicine faces additional challenges: medical text is technical, with synonyms, abbreviations, and Latin equivalents, and 3D context does not transfer easily from 2D pretraining [20,21,22]. Several systems nonetheless demonstrate strong results: LViT injects text into a U-Net-like backbone [20], the CLIP-Driven Universal Model guides segmentation of twenty-five organs and six tumor types [19], BiomedParse handles segmentation, detection, and recognition for eighty-two object types across nine modalities from text [21], BiomedParse-V extends to volumetric data [22], SAT targets general text-prompted radiology [92], and ZePT performs zero-shot pan-tumor segmentation via CLIP-based query disentangling [93].

These systems show that text prompts can replace many manual prompts in medical segmentation. They also show that natural-language interfaces can support broader use cases such as report grounding, education, and clinical decision support [21,24,94]. The remainder of this survey looks more closely at how these models are built, how they are adapted to clinical data, and where they still fall short.

## 4. Method Taxonomy

**Figure 3:**
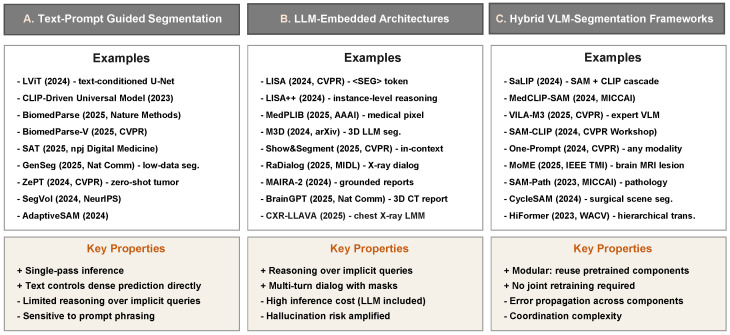
Taxonomy of vision-language foundation models for medical image segmentation. We split the literature into three categories: text-prompt-guided segmentation models, LLM-embedded architectures, and hybrid frameworks.

**Figure 4:**
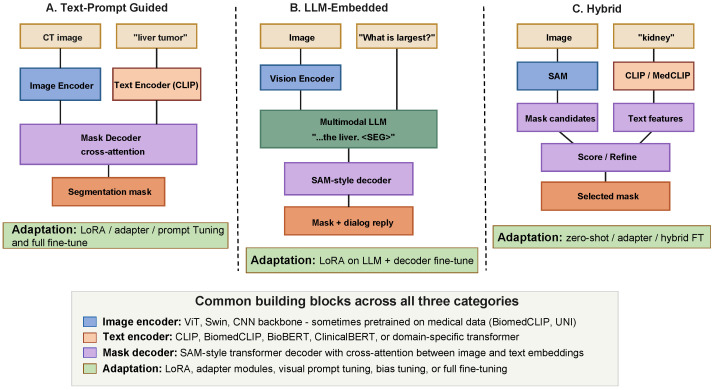
Architectural patterns for the three categories of vision-language segmentation models. Text-prompt-guided models inject text embeddings into a unified architecture. LLM-embedded models route text through a multimodal language model that produces a segmentation token. Hybrid models couple separate vision-language and segmentation components.

We propose a taxonomy with three categories: text-prompt-guided models, which inject text embeddings into a segmentation network; large-language-model-embedded architectures, in which a multimodal language model emits a token that is decoded into a mask; and hybrid frameworks, which loosely couple vision-language pretraining with a separate segmentation backbone. Figure 3 illustrates the taxonomy and Figure 4 compares the three architectural patterns. Many recent models combine elements of more than one category [21,95,96]; we assign each to its primary category by how text influences the final mask. Table 2 comprehensively compares fifty-three methods across six categories (SAM-based, text-prompt-guided, LLM-embedded, hybrid frameworks, specialised foundation models, and deep-learning baselines); the three taxonomy categories are a subset, with the others included for context.

### 4.1. Text-Prompt-Guided Segmentation

Text-prompt-guided segmentation models use text embeddings as conditioning signals inside a segmentation network: a pretrained text encoder such as CLIP, BioClinicalBERT, or a domain-specific transformer [12,15,51,52] produces an embedding that is fused with image features at one or more network stages to yield a mask [95], with training on triples of image, mask, and text description.

LViT was one of the first medical models in this category [20]: a U-Net backbone with bottleneck cross-attention that fuses clinical-note text with image features, evaluated on chest X-ray COVID-19 lesions with clear gains over text-free baselines. TGANet introduced text-guided attention for polyp segmentation, and Lee et al. used text-guided cross-position attention to refine spatial attention [97]. Adhikari et al. combined synthetic images with vision-language segmentation in echocardiography [98]. CRIS, originally for natural images, was an early CLIP-driven referring segmentation model that influenced many medical adaptations [90]. The CLIP-Driven Universal Model scales text-guided segmentation: a frozen CLIP encoder produces per-class embeddings that serve as queries in the mask decoder, with a Swin Transformer image backbone and masked back-propagation to handle partially labeled data, yielding one model that covers twenty-five organs and six tumor types [4,19]. Liu et al. extended it with dynamic class addition [99]. BiomedParse generalizes the text-prompt-guided paradigm to nine modalities and eighty-two object types [21]. Built on SEEM, its transformer decoder consumes image features and text embeddings [91], trained on six million GPT-4-harmonized triples from forty-five datasets [11], reaching a median Dice score above 90% and outperforming bounding-box methods on irregular shapes; an independent discriminator filters slices lacking the prompted object. BiomedParse-V extends this to volumetric data via fractal 2.5D encoding [22], MedSegX targets open-world unseen object types [100], and SAT trains text-prompted radiology segmentation on more than seventy CT/MRI/PET datasets, matching task-specific networks while supporting flexible prompts [92].

Several models add more sophisticated text fusion. Cross-Modal Conditioned Reconstruction trains the text-image fusion module by reconstructing masked image regions from text [95], and TGCA-PVT uses cross-position attention with a pyramid vision transformer backbone [97]. Transformer-guided multi-scale fusion (ScaleFusionNet) and dense encoder-decoder designs provide strong baselines for skin lesion and dermatological segmentation [101,102]. A practical advantage is deployment in text-driven workflows: a radiologist can dictate a target, and the model can produce the mask, though mask quality depends on prompt quality. The next category adds reasoning through large language models.

### 4.2. LLM-Embedded Architectures

Large language model embedded architectures couple a multimodal large language model with a segmentation decoder: the language model handles the conversation and emits a special token whose hidden state encodes the segmentation target, which the decoder converts into a mask. This lets the system handle implicit queries that require reasoning, world knowledge, or multi-step instructions [23,24]. LISA established the embedding-as-mask paradigm [23]: a special <SEG> token is added to a multimodal language model, and its hidden embedding is passed to a SAM-style mask decoder alongside image features, trained end-to-end so the model answers reasoning queries (e.g., “which object can hold liquid”) with both text and a mask. LISA++ added instance-level reasoning and multi-turn dialog [24]. For biomedicine, several models adapt this paradigm. MedPLIB applies the LISA-style architecture with SAM-Med2D as the mask backbone and is trained on the MeCoVQA region-text dataset [103]. M3D and Med-2E3 bring three-dimensional reasoning segmentation to multimodal language models, the latter injecting 2D priors [25,104]. RadFM and CheXagent provide radiology backbones that can be coupled with segmentation heads [54,105], and SAM 2 adds a memory mechanism for image and video segmentation [106].

Two design choices matter: whether the language model is frozen (preserving general knowledge) or fine-tuned (gaining medical precision but risking drift), with LoRA-based adaptation a common compromise [25,45,103]; and the fusion point, from a single token’s hidden state (LISA) to multiple tokens or the full output sequence [95,107]. Computational cost is a practical concern: running a multimodal language model per query can exceed several seconds, acceptable offline but limiting interactive use, which motivates distillation and lightweight backbones [71,103,108].

A further concern is hallucination: the language model can emit plausible but wrong text alongside the segmentation token, yielding an inaccurate mask or one that references an absent object [109,110]. Uncertainty calibration, fact-grounded preference optimization, and presence verification reduce these failures [22,111,112], which we revisit in Section 9.

### 4.3. Hybrid Frameworks

Hybrid frameworks loosely couple a pretrained vision-language model, which supplies semantic information from text, with a separate segmentation model that produces masks; the coupling can occur at the input, intermediate, or output stage [113,114].

Input-stage coupling converts text into prompts for a segmentation model: SaLIP cascades SAM and CLIP, generating candidate masks with SAM and scoring them against the text prompt with CLIP, requiring no extra training and supporting optional test-time adaptation [114]. Intermediate coupling injects vision-language features into the segmentation model. MedCLIP-SAM aligns medical images and text with MedCLIP, then uses the attention maps to generate point prompts for SAM and refines the resulting masks with text-aligned features [113]. Bio2Vol adapts BiomedParse to volumetric data through dual-rate sampling and cross-slice attention while preserving its pretrained 2D capabilities. Output-stage coupling validates or refines masks: CLIP text-image similarity filters false positives [21,22], language models generate explanations for review [94], and model-agnostic refiners fit into hybrid pipelines [115]. Unified lightweight frameworks can serve multiple clinical sites in multi-task, multi-center settings [116]. Overall, the hybrid approach reuses the strengths of existing pretrained models without retraining, allows modular updates as better components appear, and spans many modalities and tasks, at the cost of integration complexity and possible error propagation between components [106,117].

Recent work explores tighter integration. SAM-CLIP merges SAM and CLIP parameters into one model handling segmentation and zero-shot classification [118], while others distill a teacher VLM into a student segmenter [89]. CycleSAM uses cycle-consistent feature matching for few-shot surgical scenes [117], HiFormer supplies hierarchical multi-scale transformer features [119], SAM-OCTA targets retinal OCTA [120], and VILA-M3 injects medical expert knowledge [96]. These approaches blur the line between hybrid and integrated architectures, as surveyed for biomedical segmentation by Lee et al. [35]. Many of the best systems on recent challenges use hybrid designs. At CVPR 2025, the Foundation Models for Text-guided 3D Biomedical Image Segmentation Challenge was won by an enhanced version of BiomedParse that combines text-prompted segmentation with a separate volumetric encoder [22]. Similar designs appear in the top entries to other recent challenges [80]. SegFM3D extends 3D foundation models for universal medical image segmentation [80]. Wu and Xu demonstrated universal one-prompt medical image segmentation across organs and tumors [108]. Segment Any Medical Object proposes a generalist segmentation model that supports diverse anatomical structures and lesion types under a single framework.

The category boundaries are not sharp: BiomedParse is primarily text-prompt-guided but adds a meta-object classifier that refuses absent prompts, an LLM-like trait without a full language model [21], LISA is the prototypical LLM-embedded design yet decodes through a SAM-style mask head [23,24], and hybrids such as SaLIP and MedCLIP-SAM combine SAM and CLIP without retraining [113,114]. A useful organizing axis is therefore where text enters the decision as shown in Figure 4, inside a unified architecture, through a language-model token, or via a separate component, which in turn shapes model size, cost, and data needs: BiomedParse has roughly 100 million parameters and trains on image-mask-text triples, whereas LISA adds a 7–13B language model and instruction-tuning data, and hybrids reuse pretrained components without joint training [21,23,92]. Table 2 lists these methods with their reported performance.

**Table 2:** Overview of vision-language and segmentation foundation models for medical images. The table covers fifty-three methods from 2021 to 2026, grouped into six categories. Reported Dice values are the peak values stated by the original authors on their own benchmarks. These Dice scores are not directly comparable and do not constitute a ranking, because they were obtained on different datasets, modalities, tasks, label definitions, and evaluation protocols; they are listed only to indicate the order of magnitude of reported performance within each method’s own setting. A dash (-) indicates that a single Dice score was not reported.

Method	Year	Architecture	Modality	Datasets	Dice	Adaptation
*SAM-Based Models*						
SAM [9]	2023	ViT-H + prompt enc.	Natural images	SA-1B (1B masks)	-	Pretrain
MedSAM [43]	2024	SAM + medical FT	Multi-modal	1.5M med. image pairs	0.85	Full FT
SAM-Med2D [72]	2023	SAM + adapter	10 modalities	4.6M imgs, 19.7M masks	0.83	Adapter
SAM-Med3D [73]	2023	Native 3D SAM	Volumetric	21K imgs, 131K masks	0.78	Full FT
SAMed [85]	2023	SAM + LoRA	Multi-organ CT	Synapse BTCV	0.82	LoRA (0.1%)
Med-SA [76]	2025	SAM + Adpt. + LoRA	5 modalities	17 tasks	0.84	Adpt. + LoRA
AdaptiveSAM [83]	2024	SAM + bias tuning	Surg., US, X-ray	Multiple	0.81	Bias tuning
SegVol [79]	2024	Volumetric SAM	CT	200 organs, 96K vol.	0.83	Full FT
3DSAM-adapter [84]	2024	SAM 2D→3D adapt.	CT (tumour)	LiTS, KiTS, pancreas CT	0.86	Adapter
SAM-OCTA [120]	2025	SAM + OCTA prompt tuning	Retinal OCTA	ROSE, OCTA-500	0.87	Full FT
SAM2LoRA [88]	2025	SAM 2 + LoRA	Retinal fundus	11 datasets	0.93	LoRA (<5%)
MedSAM2 [77]	2025	SAM 2 + medical FT	Image + video	Multi-modal	0.86	Full FT
MedSAM3 [87]	2025	SAM 3 + LoRA	Multi-modal	Concept-aware	0.84	LoRA
EmbedMedSAM [121]	2025	SAM embed. + edge optim.	Multi-modal	Resource-limited settings	0.82	Adapter
*Text-Prompt-Guided Models*						
LViT [20]	2024	U-Net + text fusion	Chest X-ray	QaTa-COV19	0.83	Full FT
Cross-modal CR [95]	2024	Cross-modal recon. CLIP	CT, MRI	Multiple organ datasets	0.84	Full FT
CLIP-Driven UM [19]	2023	CLIP queries + Swin	Abdominal CT	BTCV, LiTS, KiTS	0.86	Full FT
Universal VLM [99]	2024	Extensible CLIP + dec.	Abdominal CT/MRI	BTCV, 15 organs	0.87	PEFT
ZePT [93]	2024	CLIP query disentangle	Pan-tumour CT	Multi-source	0.77	Self-prompt
BiomedParse [21]	2025	SEEM + GPT-4 harm.	9 modalities	BiomedParseData (6M)	0.94	Full pretrain
BiomedParse-V [22]	2025	FVE + ISD module	CT, MRI, micro.	CVPR 2025 challenge	0.86	Full pretrain
MedSegX [100]	2025	Generalist FM + open vocab	Multi-modal	100+ datasets	0.85	Full pretrain
SAT [92]	2025	CLIP + transf. dec.	Radiology	70+ datasets	0.84	Full FT
*LLM-Embedded Architectures*						
LISA [23]	2024	MLLM + 〈SEG〉 tok.	Nat. + reasoning	ReasonSeg, refCOCO	-	Full FT (LLM)
LISA++ [24]	2024	LISA + inst. reasoning	Nat. + medical	Extended ReasonSeg	-	Full FT
ChatRadio-Valuer [122]	2025	LLM + rad. impression dec.	Chest X-ray	Multi-inst. CXR	-	Full FT
MedPLIB [103]	2025	MLLM + SAM-Med2D	Multi-mod. med.	MeCoVQA	0.81	LoRA + Adpt.
M3D [25]	2025	3D MLLM + decoder	3D CT	M3D-Seg	0.79	Full FT
Show & Segment [123]	2025	In-context MLLM + dec.	Multi-modal med.	12 diverse datasets	0.83	Zero-shot
*Hybrid and Other Frameworks*						
MedCLIP-SAM [113]	2024	MedCLIP + SAM	Multi-modal	Multiple	0.80	Hybrid
SaLIP [114]	2024	SAM + CLIP cascade	Multi-modal	Multiple	0.74	Zero-shot
VILA-M3 [96]	2025	VLM + medical expert know.	Multi-modal	BTCV, LiTS, BraTS	0.86	PEFT
SegFM3D [80]	2025	3D foundation model	Multi-modal 3D	Multi-source	0.83	Pretrain
*Specialized Foundation Models*						
MoME (lesion) [124]	2025	Mixture of mod. experts	Brain MRI lesions	Multi-source MRI	0.80	Full FT
UniverSeg [125]	2023	Few-shot universal	16 modalities	MegaMedical	0.72	Few-shot
GenSeg [126]	2025	Diffusion gen. + seg.	Multi-modal	Ultra low-data regimes	0.81	Hybrid gen.
SegMamba-V2 [127]	2026	Mamba SSM 3D long-range	Volumetric CT/MRI	Multi-organ 3D	0.88	Full FT
TotalSeg. [39]	2023	nnU-Net based	CT (104 structs.)	1204 CTs	0.94	Full train
TotalSeg. MRI [40]	2025	Seq.-independent	MRI (multi-organ)	616 MRI + 527 CT	0.84	Full train
BrainSegFounder [128]	2024	Self-sup. 3D ViT	Brain MRI	Multi-source neuroimaging	0.91	PEFT
SAMUS [81]	2024	SAM + US adapt.	Ultrasound	Multi-source US	0.80	Adapter
SegAnyBone [82]	2024	SAM + bone FT	MRI bones	Multi-seq. MRI	0.82	Full FT
Self-imp. FM [129]	2025	Generative FM + self-imp.	CT, MRI, X-ray	Multi-organ, multi-modal	0.85	Full pretrain
LCTfound [130]	2026	Lung CT ViT FM	Chest CT	LIDC-IDRI, NLM, LUNA16	0.89	Full pretrain
Merlin [131]	2026	CT VLM + report gen.	Chest CT	Radiology reports + seg.	-	Full pretrain
Decipher-MR [132]	2026	3D MRI VLM encoder	Multi-seq. MRI	Diverse MRI tasks	-	Full pretrain
CT-CLIP [59]	2026	Volumetric CLIP	Chest CT	CT-RATE (50K)	-	Pretrain
*Deep Learning Baselines (CNN/Transformer, no text prompt)*						
Confidence-SS [133]	2025	CNN-Trans. semi-sup.	Skin lesion	ISIC 2016, PH2	0.91	Semi-supervised
H-Self-Support [134]	2026	Hierarchical self-support	Brain MRI (tumour)	BraTS 2021	0.92	Self-supervised
Dense Enc.-Dec. [102]	2021	CNN enc.-dec. skip conn.	Skin lesion	ISIC 2018, PH2	0.87	Full FT
RD2A [2]	2021	Residual dense + ASPP	Brain MRI (tumour)	BraTS 2019	0.89	Full FT
ScaleFusionNet [101]	2025	Trans. multi-scale FPN	Skin lesion	ISIC 2017/2018, PH2	0.90	Full FT
UNet-Mamba [5]	2025	UNet + Mamba-like attn.	Multi-modal	ACDC, Synapse, polyp sets	0.91	Full FT

## 5. Adaptation Strategies

Vision-language foundation models are pretrained on general or biomedical data and usually need further training to reach a specific clinical task, where the adaptation strategy governs accuracy, computational cost, and deployment flexibility. We discuss three main strategies, full fine-tuning, parameter-efficient fine-tuning, and prompt engineering, whose tradeoffs are summarized in Table 3 [45,46,135].

### 5.1. Full Fine-Tuning

Full fine-tuning updates all parameters and typically achieves the best accuracy when training data is sufficient [43,72]. MedSAM fine-tunes the SAM mask decoder while partially freezing the encoder [43], and SAM-Med2D fully fine-tunes on more than four million masks [72,78]. Common objectives include Dice, focal, generalized Dice, and Lovász-Softmax losses [136,137,138]. Full fine-tuning is costly: SAM’s ViT backbone exceeds six hundred million parameters [3], requiring large GPU memory and time (MedSAM used twenty A100 GPUs for weeks; BiomedCLIP used sixteen) [15,43], beyond the reach of most groups. It also risks catastrophic forgetting of general knowledge [139] and produces a full model copy per task. Parameter-efficient fine-tuning addresses these issues by updating only a small fraction of parameters.

### 5.2. Parameter-Efficient Fine-Tuning

**Figure 5:**
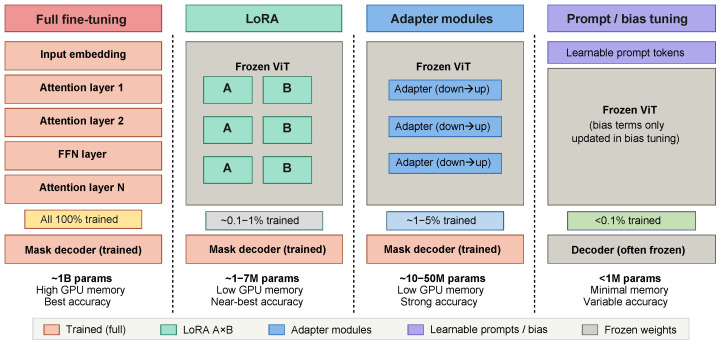
Comparison of parameter-efficient fine-tuning methods for medical foundation models. Full fine-tuning updates all parameters but has the highest cost. LoRA, adapters, and prompt tuning offer increasingly lightweight alternatives with different accuracy-cost tradeoffs.

Parameter-efficient fine-tuning (PEFT) updates only a small subset of parameters while freezing the rest, preserving original capabilities, reducing training memory, enabling many task-specific adapters, and often approaching full fine-tuning accuracy as in Figure 5 [45,46,140].

Low-Rank Adaptation (LoRA) is the most widely used PEFT method [45]: it adds two trainable low-rank matrices whose product approximates the weight update, typically under one percent of the parameters. SAMed applied LoRA to SAM’s image encoder and matched the state of the art on Synapse while updating only 0.1% of parameters [85], and SAM2LoRA applied it to both encoder and decoder of SAM 2 for retinal fundus segmentation, reaching Dice up to 0.93 with under five percent of parameters trained [88]. Adapter modules insert small trainable layers between frozen ones [46]: Medical SAM Adapter (Med-SA) combines adapters and LoRA with a Space-Depth Transpose for 2D/3D images and surpassed SAM across seventeen tasks and five modalities [76], while AdaptiveSAM tunes only bias terms and adds text-prompted segmentation [83]. Several variants extend LoRA. Conv-LoRA injects local convolutional inductive biases into the ViT encoder while preserving SAM’s segmentation knowledge, AdaLoRA allocates the rank budget adaptively across layers [141], and DoRA and NAS-LoRA refine the magnitude/direction decomposition and configuration search. Visual prompt tuning adds learnable input tokens and suits classification more than dense prediction [142], while CLIP-Adapter and Tip-Adapter attach small networks on frozen CLIP features [143]. Prompt tuning for medical segmentation was studied by Fischer et al. [135], and few-shot PEFT can be cheaper and stronger than in-context learning [140].

A 2024 empirical study of PEFT for SAM across seventeen datasets and five modalities found that the right PEFT strategy slightly outperforms prior methods, that LoRA suits small datasets while adapters suit larger diverse ones, and that fine-tuning the mask decoder matters more than the encoder for medical tasks [34].

### 5.3. Prompt Engineering

Prompt engineering exploits in-context learning without updating parameters: the user designs prompts to elicit the desired behavior. It is the cheapest and most flexible adaptation, limited mainly by prompt quality and alignment with pretraining data.

For text-prompted models, prompt design matters: BiomedParse uses GPT-4 to harmonize descriptions across forty-five datasets, and specific prompts such as “glandular structure in colon pathology” reach a median Dice score of 0.942 while vague prompts perform worse [21]. SAT-style models use structured anatomical, modality, and pathology prompts [92], and self-regulating prompts reduce forgetting during adaptation [139]. Visual prompt design also matters for SAM: point placement strongly affects quality, bounding boxes constrain better than points, and iterative prompting is the most accurate but also the most interactive [31,32,33]. Combining text and visual prompts is increasingly common: SegVol shows their combination improves accuracy on complex anatomy [79], and AdaptiveSAM integrates text with bounding-box adaptation [83]. Chain-of-thought prompting has been adapted to vision-language models: LLaVASeg uses multi-step prompts that first reason about the target, then identify attributes, then produce the mask, which helps with complex queries but adds latency [24]. Prompt reliability is an active topic: different phrasings, synonyms, and abbreviations of the same target yield different masks [21,97], and negation is hard because contrastive models tend to ignore it [69]; prompt augmentation, ensembling, and semantic-aware design help [69,92]. The best strategy depends on resources, data, and deployment scenario. Full fine-tuning gives the best accuracy with abundant data and compute; PEFT supports many tasks from one base model and suits multi-task, cross-institutional, and continual deployment, since adapters can be switched or added without disturbing the frozen base and federated learning preserves privacy [8,75]; and prompt engineering suits rapid prototyping. As summarized in Table 3, LoRA with rank 4–16 reaches accuracy within a few percent of full fine-tuning while training 1–5% of the parameters, and hybrid recipes such as SAMed mix LoRA with selective full fine-tuning [79,85]. PEFT additionally aids interpretability and reproducibility by making explicit which parameters are adapted; reported results should include random seeds, hardware, and training duration as shown in Figure 5.

## 6. Modality-Specific Models

Imaging modalities pose different challenges: CT is volumetric with strong organ contrast, MRI offers rich soft-tissue contrast across sequences, pathology yields gigapixel whole-slide images, chest radiography is fast but two-dimensional, and ultrasound is real-time but operator-dependent. We review vision-language foundation models per modality, emphasizing recent 2024–2025 work [39,40,41,105].

### 6.1. Computed Tomography

Computed tomography is the most common volumetric modality, varying in contrast phase, slice thickness, and protocol; vision-language models must handle volumetric input, anatomical variability across patients, and the relationships among adjacent organs.

The CLIP-Driven Universal Model uses CLIP text embeddings of organ names as mask-decoder queries to segment twenty-five organs and detect six tumor types, training on fourteen partially labeled CT datasets through masked back-propagation and reaching strong results on BTCV and LiTS [19,144,145]. Liu et al. extended it to handle dynamic addition of new classes without retraining [99]. TotalSegmentator covers 104 anatomical structures in CT using an nnU-Net backbone and a curated set of more than one thousand scans, with a 2025 update adding sequence-independent MRI segmentation [1,39,40]; though prompt-free, its taxonomy has informed many text-prompted models. SegVol adds text and spatial prompts for more than two hundred CT classes from ninety thousand unlabeled and six thousand labeled volumes [79], and UniverSeg generalizes across fifty-plus datasets and sixteen modalities via few-shot cross-attention with support sets rather than text [125]. CT-CLIP and Merlin brought vision-language pretraining to volumetric chest CT, training on more than fifty thousand CT volumes with paired structured reports from CT-RATE and supporting zero-shot disease detection and report generation [59,131]. M3D and Med-2E3 extended multimodal language models to general 3D CT analysis [25,104], and BiomedGPT provides a generalist backbone covering CT [146]. Recent work targets specific CT tasks: one-prompt segmentation across modalities [108], a self-improving generative model trained on heterogeneous CT and MRI [129], and UniMed-CLIP for unified image-text pretraining across modalities including CT [147].

Three-dimensional context remains a critical challenge: 2D-pretrained models lose information slice by slice. BiomedParse-V uses fractal volumetric encoding [22], while SAM-Med3D, SegVol, and SegFM3D natively process 3D inputs [73,79,80], and LCTfound adds a 2026 lung-CT foundation model [130]. SegMamba and self-adaptive Mamba-like designs offer state-space alternatives that scale to long volumetric sequences [5,127,148], and a data-efficient 3D VLM using only a 2D encoder reaches competitive 3D understanding at lower cost [149]. Still, true 3D vision-language pretraining at the scale of 2D has not yet been achieved [26,27].

CT also raises modality-specific issues. Contrast phase matters: models trained mainly on portal-venous data may underperform on other phases, motivating phase-aware conditioning [142]. Slice thickness and reconstruction kernel affect small-structure accuracy, and most models are trained on adult data, which makes PEFT valuable for pediatric use [79,129].

### 6.2. Magnetic Resonance Imaging

MRI offers excellent soft-tissue contrast through multiple sequences (T1, T2, FLAIR, DWI) that provide complementary information, so vision-language models must handle this multi-sequence nature. Brain MRI is one of the most studied applications. Swin UNETR and BrainSegFounder provide 3D backbones, the latter pretrained on large-scale neuroimaging to reduce labeling needs [6,128], and the BraTS series supplies multi-sequence benchmark data with more than two thousand labeled cases [150,151]. Residual-dense ASPP networks and hierarchical self-support learning improve multi-grade tumor segmentation under label scarcity [2,134]. Cardiac MRI is another active area: ACDC anchors chamber segmentation [152], and Christensen et al. introduced an echocardiography vision-language model for cardiac function assessment [153]. SegmentAnyBone uses text prompts to segment bones across T1, T2, PD, and STIR sequences [82], and Med-2E3 extends the 2D-enhanced 3D MLLM to MRI [104]. Prostate MRI benefits from universal models such as UniverSeg and SAM-based PEFT with minimal labeled data [34,125]. Diffusion approaches have also been explored for ambiguous MRI segmentation, including Diff-UNet for volumetric data [154,155].

MRI poses specific challenges: intensities are not standardized across scanners, datasets are smaller than CT or chest X-ray, and multi-sequence input increases memory cost [7]. Most models still process sequences independently or as channels rather than modeling their relationships, which sequence-aware designs and sequence-conditioned prompts address, and DWI remains underrepresented in pretraining [29,62]. Still, text-prompted MRI segmentation is now practical: TotalSegmentator MRI gives sequence-independent segmentation [40], Decipher-MR provides 3D MRI representations for diverse tasks [132], and hierarchical SAM decoding improves fine-grained prediction [156]. Image-translation methods bridge missing sequences [157,158], and Mamba-based state-space architectures (U-Mamba, Swin-UMamba, VMamba) offer efficient alternatives for volumetric data [159,160,161,162,163].

### 6.3. Pathology

Pathology presents gigapixel whole-slide images, so models operate on patches and aggregate across the slide while handling this scale and the rich semantic vocabulary of pathology. PLIP was an early pathology vision-language model, trained on more than two hundred thousand image-text pairs from medical Twitter to support zero-shot patch classification and retrieval [60], and Virchow scaled this to clinical-grade pathology and rare-cancer detection [63]. UNI provides strong image-only features from hundreds of thousands of whole-slide images that can be coupled with text decoders [62], Quilt-1M supplies one million image-text pairs for pathology pretraining [61], and PathAsst offers a generative conversational assistant covering localization and segmentation [64]. Deng et al. evaluated SAM zero-shot on digital pathology [74]. For text-guided pathology segmentation, BiomedParse reaches a median Dice above ninety percent on glandular structures from prompts such as “glandular structure in colon pathology” and handles irregular cellular shapes that challenge bounding-box methods [21]. Hierarchical multi-scale transformers extend to high-resolution pathology [119], while RetCCL and masked-image-modeling backbones support retrieval and stronger features [65,66]. H-optimus-0 adds a recent open-source pathology backbone with strong downstream features, and Bio2Vol enables parameter-efficient adaptation of 2D pathology models to volumetric data.

Key challenges for pathology are the large technical vocabulary and stain and color variation across institutions; privacy-preserving and federated learning offer routes to cross-institutional training without exposing patient data [164,165,166,167].

### 6.4. Chest Radiography

Chest radiography has the most mature vision-language ecosystem because large datasets pair images with reports: MIMIC-CXR alone contains more than 370,000 images with free-text reports [168], complemented by CheXpert, PadChest, and VinDr-CXR. Tanida et al. introduced interactive, region-guided report generation [94]. GLoRIA, BioViL, and BioViL-T refined contrastive pretraining for chest radiography, achieving zero-shot pathology detection comparable to radiologists [16,17,18], and RaTEScore provides a radiologist-aligned metric for report generation [169]. For chest X-ray segmentation, LViT uses report excerpts as text prompts for COVID-19 lesions [20], and CXR-LLAVA adds interpretation and grounded reporting [41]. CheXagent provides grounded report generation and a multi-task chest X-ray foundation model [105], while RaDialog, interpretable concept-bottleneck reporting, and ChatRadio-Valuer extend dialog and impression generation [122,170,171,172]. CheXstray supports drift detection in deployed imaging AI [173], and federated split vision transformers address cross-institutional COVID-19 CXR learning [167].

Bias is a particular concern: some chest X-ray models underperform on under-represented populations [174,175], foundation models are vulnerable to imaging artifacts [176], and mitigation strategies plus fact-grounded preference optimization aim to address these failures [112,177].

### 6.5. Ultrasound

Ultrasound is portable, real-time, and radiation-free but noisier and more operator-dependent than CT or MRI, with quality varying by probe position and gain, which makes it difficult for vision-language models.

SAMUS adapts SAM to clinical ultrasound with parameter-efficient tuning and ultrasound-specific data, outperforming general SAM on multiple tasks [81], while EchoCLIP brought contrastive vision-language pretraining to echocardiography, training on more than one million cardiac ultrasound videos with expert interpretations for strong zero-shot cardiac-function assessment [153]. For obstetric and abdominal ultrasound, recent models target specific structures using benchmark datasets such as BUSI (breast) and thyroid nodule sets. Adhikari et al. used diffusion-synthesized data to enhance echocardiography segmentation [98], and SAM-OCTA extends prompted segmentation to retinal angiography [120]. Ultrasound remains hard because large image-text datasets are scarce, image quality is operator-dependent, and real-time inference is often required. Even so, adapted SAM and vision-language models now give clinically useful ultrasound segmentation, with active work on portable, point-of-care integration [56,178,179].

## 7. Clinical Applications

Clinical applications span the diagnostic and therapeutic workflow. We focus on three high-impact areas, organ segmentation, tumor segmentation, and radiotherapy planning, with others including surgical planning, image-guided procedures, and conversational diagnostic AI [8,178,179].

A caveat applies throughout this section: the great majority of the models discussed below are research prototypes evaluated retrospectively on public benchmarks, not tools cleared for clinical use. Among the systems reviewed, only broad-coverage segmentation tools such as TotalSegmentator are in routine research and some clinical workflows, and very few text-prompted or LLM-embedded models have undergone prospective, multi-site, or regulatory validation. We therefore describe the clinical tasks these models target as research potential, and separate this from demonstrated clinical deployment; the maturity of each main model is summarized in the quality-assessment table later in this review.

### 7.1. Organ Segmentation

Whereas traditional pipelines trained a separate model per organ and modality, text-prompted universal models now segment dozens to hundreds of organs from a single network: the CLIP-Driven Universal Model (25 organs) [19], SegVol (200+ categories with text and spatial prompts) [79], and the example-based UniverSeg [125], evaluated on benchmarks such as the Medical Segmentation Decathlon and AMOS. TotalSegmentator and its 2025 MRI extension are widely adopted in clinical and research workflows, reducing manual contouring [39,40], and extensible universal models handle dynamic class addition for evolving taxonomies [99]. For specific organs, flexible interfaces include SegmentAnyBone for multi-sequence bone MRI [82], self-improving generative models across modalities [129], MRI lesion models [124], and in-context universal segmentation [123], guided by emerging ethical frameworks [180].

A practical advantage is rapid deployment: a new task can often be addressed by writing a text prompt rather than training a model, though accuracy may trail a dedicated model when the class was unseen in pretraining [31].

### 7.2. Tumor Segmentation

Tumors vary widely in shape, size, location, and boundary clarity, which text-driven specification helps address. ZePT performs zero-shot pan-tumor segmentation via CLIP-based query disentangling and self-prompting [93], the CLIP-Driven Universal Model covers six tumor types [19], and BiomedParse handles multiple tumor types across nine modalities with strong results on irregular shapes [21]. Brain tumor segmentation is a long-standing application tracked by the BraTS series [150,151], with Swin UNETR and BrainSegFounder as 3D backbones [6,128]. Vision-language prompts can specify tumor type and sequence, and diffusion-based methods including Diff-UNet have been applied to ambiguous tumor boundaries [154,155]. For liver tumor segmentation on LiTS [145], foundation-model-guided semi-supervised CT segmentation works in resource-constrained settings [181], and confidence-weighted CNN-Transformer semi-supervision helps when labels are scarce [133]. The 3DSAM-adapter targets promptable volumetric tumors [84], while ultrasound foundation models and expert-knowledge VLMs extend coverage [96,182]. Kidney tumors use the KiTS benchmarks [183,184,185], where the state of the art combines foundation backbones with task-specific fine-tuning, and skin lesion work builds on ISIC and HAM10000 [101,102,133]. Ophthalmic VLMs covering hundreds of fundus diseases extend the paradigm to retinal pathology [120]. Tumor heterogeneity across patients, time points, and protocols remains a core difficulty; cross-modal contrastive losses and clinical metadata help [95], and clinical-environment simulators support rigorous evaluation [186]. Integration with single-cell or molecular foundation models such as scGPT and Geneformer could unlock new tumor-analysis modalities [187,188].

### 7.3. Radiotherapy Planning

Radiotherapy planning requires precise, consistent contouring of target volumes and organs at risk, which is time-consuming manually. Early deep-learning systems delineated head-and-neck organs at risk with dedicated per-anatomy models [36,37,38]; vision-language foundation models now offer a unified, text-driven alternative. Broad-coverage models such as TotalSegmentator and the CLIP-Driven Universal Model serve as starting points for organ-at-risk delineation and adapt to site-specific contouring with limited data [19,39], and in-context universal segmentation fits radiotherapy use cases [99,123].

Clinical adoption is gradual: regulatory validation across populations and the interpretability of foundation models remain concerns [171,180], even as some vendors incorporate foundation-model components into planning systems. Systematic reviews of healthcare LLM testing and reports of GPT-4 on complex cases inform deployment expectations [189,190,191].

## 8. Evaluation and Datasets

Evaluation must capture both region overlap and boundary accuracy. Standard metrics are the Dice score, Intersection over Union, and the 95th-percentile Hausdorff Distance, each with known limitations [47,48,49,50]; Table 4 lists their properties and typical use.

### 8.1. Evaluation Metrics

The Dice score (twice the intersection over the summed volumes) is the most reported overlap metric but is sensitive to class imbalance [49,136]. IoU (the Jaccard index) is a more conservative overlap measure [47]. Boundary metrics complement overlap: the 95th-percentile Hausdorff Distance (HD95) is widely used for organs at risk, while Average and Normalized Surface Distance report mean boundary error and the fraction of boundary within tolerance [49,50].

For multi-focal disease, lesion-wise Dice evaluates each lesion separately before aggregating, which is more meaningful than global Dice, and lesion-level sensitivity and specificity together with detection hits and false alarms per image suit screening [183,184,185].

Text-prompted segmentation needs additional measures: the median Dice across prompts captures consistency of response to paraphrases of the same target, object-recognition accuracy and the negative-prediction rate test whether the model correctly detects or refuses an absent object (BiomedParse uses a Kolmogorov–Smirnov test for invalid prompts), and RaTEScore evaluates the text output of LLM-embedded systems that produce both masks and reports [21,169].

Multi-class scores can be aggregated by mean, frequency-weighted mean, or worst-case, each with different clinical implications. Maier-Hein et al. and Reinke et al. stress selecting metrics by clinical question and reporting all relevant numbers with uncertainty rather than a single headline value [47,48], and loss design (focal, generalized Dice, Lovász-Softmax) shapes how metrics behave during training [136,137,138].

### 8.2. Benchmark Datasets

Table 5 summarizes widely used benchmark datasets by modality; below we highlight those most relevant to vision-language segmentation.

Abdominal CT relies on BTCV, AMOS, FLARE, LiTS, KiTS, and the Medical Segmentation Decathlon [144,145,183,184,185]. Brain and cardiac MRI use the BraTS series and ACDC [150,151,152], and echocardiography adds EchoNet/EchoCLIP [153]. Chest X-ray benefits from MIMIC-CXR, CheXpert, PadChest, NIH ChestX-ray14, and VinDr-CXR [168], while pathology draws on Quilt-1M, PMC-CLIP, GLaS, and CAMELYON [53,61,65,66]. Ultrasound and dermatology use BUSI, ISIC, and HAM10000.

For text-prompted segmentation specifically, BiomedParseData provides six million image-mask-description triples harmonized from forty-five datasets with GPT-4 [21], the CVPR 2025 challenge adds 3D benchmark data [22], SAT curates text-annotated radiology from over seventy datasets [92], and CT-RATE pairs chest CT volumes with reports [59,192]. General-domain sets such as COCO and Open Images also support pretraining, and clinical-environment simulators aid dynamic evaluation [186].

Data leakage between web-scale pretraining and public benchmarks is a growing concern, so studies should disclose pretraining data and evaluate on held-out clinical data [174,175]. Privacy-preserving and federated learning enable cross-institutional training without raw-data exchange [164,165,166], and harmonizing heterogeneous datasets requires consistent ontologies such as those used by BiomedParseData and SAT [21,92].

## 9. Challenges and Future Directions

**Figure 6:**
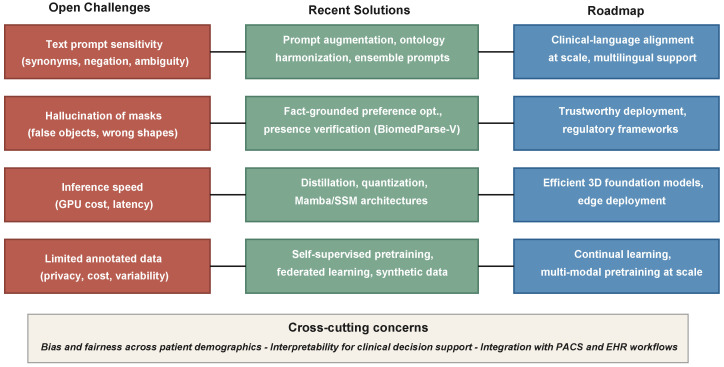
Challenges and research roadmap for vision-language foundation models in medical image segmentation. The figure shows the four open challenges (prompt sensitivity, mask hallucination, inference speed, and limited annotated data), each paired with recent mitigations and longer-term roadmap directions, together with cross-cutting concerns spanning bias and fairness, interpretability, and clinical workflow integration.

Despite rapid progress, vision-language foundation models for medical segmentation face open challenges that also define the research agenda. We group them into four themes, prompt dependence and trustworthiness, efficiency and 3D scaling, data efficiency, and clinical integration, pairing each challenge with the directions most likely to address it. Figure 6 summarizes the challenges and roadmap. Cross-cutting concerns include privacy, fairness, regulatory approval, and interpretability [164,174,175,177,180].

### 9.1. Study-Quality Assessment

To complement the descriptive comparison in Table 2, Table 6 assesses the evidence quality of the main models along dimensions that matter for a review: peer-review status, training-data scale, external or multi-site validation, the risk that public evaluation benchmarks overlap large or web-scale pretraining corpora (data leakage), open code and weights, and clinical validation. Three limitations recur. First, almost all evaluation is retrospective on public benchmarks, and prospective or multi-site validation is rare, so reported Dice scores are an upper bound on real-world performance. Second, the largest text-prompted models are trained on datasets harmonized from many public sources such as BiomedParseData, SAT, CT-RATE, which raises a real risk of overlap between pretraining data and evaluation benchmarks; few papers explicitly audit this overlap or evaluate on fully held-out clinical data, echoing the data-leakage concern raised in Section 8. Third, peer-review status is mixed: several widely cited methods remain preprints, and their results should be weighted accordingly. These observations reinforce the need for disclosure of pretraining data and evaluation on independent clinical cohorts.

### 9.2. Prompt Dependence and Trustworthiness

Vision-language models depend critically on text prompts: the same target (“liver tumor,” “hepatic mass,” “neoplasm involving segment VIII”) can be phrased many ways that yield different masks, which makes evaluation prompt-dependent, complicates cross-institution use, and degrades on non-English text [21,56,71,97]. Mitigations include prompt augmentation during training, ensemble prompting at inference, ontology-based semantic-aware prompt design, and self-regulating prompts that reduce forgetting during adaptation [21,92,139]. Two weaknesses persist: contrastive models tend to ignore negation (“liver without tumor” resembles “liver with tumor”) [69], and compositional prompts that combine several constraints are handled only partially, even by reasoning models such as LISA [23,24]. Closing the gap to rich clinical language (location, morphology, density, enhancement) and to multilingual use remains open, with BiomedParseData and CT-RATE moving in this direction [59,178].

Hallucination is the most safety-critical issue: a model may segment absent objects, miss present ones, or produce wrong boundaries, with consequences ranging from unnecessary biopsy to incorrect radiotherapy dose [38,109,110,111,176]. Mitigations include presence verification (BiomedParse’s independent segmentation discriminator), fact-grounded preference optimization with physician feedback, diagnosis-guided bootstrapping, and confidence calibration via temperature scaling, ensembles, or Bayesian methods [22,47,112]. Reliable inference-time detection, through confidence, visual-textual consistency, or retrieval-augmented verification, remains unsolved yet essential for clinical use, alongside validation across populations and evolving regulatory frameworks [180,189,190,191].

### 9.3. Efficiency and 3D Scaling

Many clinical workflows require segmentation in seconds, yet vision-language models often have hundreds of millions to billions of parameters [3,15]; SAM’s largest encoder alone exceeds six hundred million parameters, and LLM-embedded models add a language model per query [9,23,103]. Distillation to smaller students (MobileSAM, FastSAM, EfficientSAM), quantization, one-prompt segmentation, model-agnostic refinement, and lightweight adapters reduce cost [67,68,108,115,142,143], while Mamba-style state-space models give linear-time alternatives for long volumetric sequences [127,148,159,160,162,163]. Three-dimensional context is the deeper bottleneck: 2D-pretrained models lose information slice by slice, and 2.5D cross-slice attention or native 3D models trade speed for accuracy [22,73,193]. Edge deployment at the point of care is enabled by mobile variants, EmbedMedSAM, efficient bedside inference, and two-stage screening-then-refinement pipelines [80,108,121,124,153]. Looking forward, native multimodal pretraining that includes volumetric data, time series, and structured metadata, exemplified by CT-CLIP, BiomedParse-V, and UniMed-CLIP, is needed but still lags 2D pretraining in scale [59,147,161], and genomic and molecular foundation models suggest broader integration [187,188].

### 9.4. Data Efficiency

Annotated data is scarce because expert contouring is costly, variable, and constrained by privacy [164]. Vision-language and self-supervised pretraining on weakly labeled image-text pairs (PLIP, Quilt-1M, CT-RATE) and unlabeled images (DINO, DINOv2, masked image modeling) reduces this dependence [59,60,61,62,67,68,128]. Few-shot PEFT, hierarchical self-support learning, synthetic data from diffusion models (GenSeg) and modality translation, federated learning across institutions, and uncertainty-guided active learning all extend labeled data further, though synthetic data may inherit generator biases [47,126,134,140,143,157,158,165,166]. Even so, the gap between training-data scale and clinical need remains large, motivating data-efficient learning, cross-institution transfer, and drift monitoring [35,123,173].

### 9.5. Clinical Integration and Outlook

Clinical adoption requires operating within PACS, RIS, and EHR systems, respecting privacy, and integrating with decision support and reporting, with drift detection and clinical-environment simulators supporting safe operation [70,94,170,171,186]. Patient-specific priors and federated continual updates (with versioning and audit trails), multilingual models for global health, and hybrid pairings of general models with task-specific components and open releases (MedSAM, BiomedCLIP, BiomedParse, LLaVA-Med) will broaden access [10,15,21,42,43,71,113,118]. Emerging 2025–2026 directions include text-prompted volumetric models competitive with task-specific 3D networks, generative augmentation, reasoning over clinical implications, specialty-specific models, and integration of non-imaging data [22,122,194]. As the boundaries between segmentation, detection, classification, and generation blur, clinical impact will hinge on safety, fairness, equitable access, and evolving FDA/EMA oversight; generalist and conversational diagnostic AI and joint imaging-molecular models point toward foundation models becoming standard tools across biomedical research and practice [8,179].

## 10. Conclusions

This survey reviewed vision-language foundation models for medical image segmentation: the technical background, a three-part taxonomy (text-prompt-guided, large-language-model-embedded, and hybrid frameworks), adaptation strategies, and the literature organized by imaging modality and clinical application, together with evaluation metrics and benchmark datasets. Progress has been rapid; the period from 2024 to 2026 produced more advances than the previous decade, with models such as BiomedParse, MedSAM2, and SAT now segmenting many modalities and structures from text prompts while LoRA and adapters make deployment practical. We identified four open challenges, prompt dependence, hallucination, inference speed, and data scarcity, and set out a research roadmap toward trustworthy, well-integrated deployment spanning multimodal pretraining and clinical integration. The potential clinical payoff is substantial—lower segmentation time and cost, more consistent results across institutions, and new applications such as grounded radiology reporting and conversational decision support—but most of these benefits remain to be demonstrated in prospective clinical validation rather than retrospective benchmarks.

## Figures and Tables

**Table 1:** Comparison of recent surveys related to vision-language foundation models in medical imaging. Our work focuses specifically on segmentation and on the rapid developments in 2025 and 2026.

Survey	Year	Focus	Coverage	Contribution/Limitation
Shamshad et al. [7]	2023	Transformers in medical imaging	Transformer architectures	Pre-foundation-model era, no VLM focus
Zhao et al. [29]	2023	CLIP in medical imaging	Classification and retrieval	Limited segmentation coverage
Mazurowski et al. [31]	2023	SAM in medical image analysis	Experimental study, many datasets	Visual-prompt SAM only, no language component
He et al. [32]	2023	SAM benchmark in medical images	Accuracy across 12 datasets	SAM-only, no text-prompted segmentation
Cheng et al. [33]	2023	SAM prompt-mode study	Point, box, and mask prompting	Visual prompts only, no VLM coverage
Gu et al. [34]	2024	Foundation-model segmentation best practices	SAM adaptation, empirical	Centred on SAM, not language-guided
Lee et al. [35]	2024	Foundation models for medical image segmentation	Zero-shot evaluation	Narrative review, no VLM taxonomy
Sun et al. [28]	2025	Multimodal foundation models for diagnosis and treatment	Applications, challenges, directions	Broad clinical scope, not segmentation-specific
Khan et al. [26]	2025	Foundation models in medicine	Comprehensive survey	Broad coverage, less depth on segmentation taxonomy
Wu et al. [27]	2025	VLFM for 3D medical imaging	Report generation focus	Limited segmentation scope
Lurz et al. [30]	2026	Foundation models in medical image segmentation	Segmentation foundation models	Short overview, limited language-guided depth
This work	2026	VLM FM for medical segmentation	Architectures, adaptation, modalities, applications	Language-guided taxonomy with focus on 2025–2026 work

**Table 3:** Comparison of adaptation strategies for vision-language foundation models. The choice depends on dataset size, deployment scenario, and accuracy requirements.

Strategy	Trainable	GPU Mem.	Accuracy	Storage	Example
Full fine-tuning	100%	Very high	Highest	Full model copy	MedSAM
LoRA	0.1–1%	Low	Near best	Small adapter	SAMed
Adapter modules	1–5%	Low	Strong	Small modules	Med-SA
Bias tuning	<0.5%	Very low	Good	Bias deltas only	AdaptiveSAM
Visual prompt tuning	<0.1%	Minimal	Variable	Prompt tokens	VPT variants
Prompt engineering	0%	None	Variable	Text only	BiomedParse
Conv-LoRA	0.5–2%	Low	Strong	Small modules	Conv-LoRA SAM
NAS-LoRA	1–3%	Low	Strong	Searched arch	NAS-LoRA
DoRA	0.2–1%	Low	Strong	Magnitude+dir.	DoRA variants

**Table 4:** Common evaluation metrics for medical image segmentation. The choice of metric depends on the clinical question and the structure that is segmented.

Metric	Range	Property	Best Used for
Dice Score	[0, 1]	Overlap, small-structure sensitive	Volume overlap reporting
IoU (Jaccard)	[0, 1]	Conservative overlap	Detection metric comparison
Hausdorff Distance	[0, ∞) mm	Worst-case boundary error	Outlier sensitivity studies
95% Hausdorff (HD95)	[0, ∞) mm	Percentile boundary error	Radiotherapy organs at risk
Average Surface Dist.	[0, ∞) mm	Mean boundary error	Boundary quality reporting
Normalized Surface Dist.	[0, 1]	Boundary within tolerance	Clinically acceptable boundary
Lesion-wise Dice	[0, 1]	Per-lesion overlap	Multi-focal disease
Sensitivity/Recall	[0, 1]	Detection rate	Screening applications
Specificity	[0, 1]	False-positive rate	Specificity-critical tasks
Recognition Accuracy	[0, 1]	Object presence detection	Text-prompted segmentation

**Table 5:** Summary of widely used benchmark datasets for medical image segmentation, organized by modality. Recent datasets such as BiomedParseData and CT-RATE specifically support vision-language pretraining and text-prompted segmentation.

Dataset	Year	Modality	Size	Annotation Type	Primary Use
BTCV (Synapse)	2015	CT	30 scans, 13 organs	Voxel mask	Multi-organ benchmark
AMOS22	2022	CT, MRI	500 CT + 100 MRI, 15 organs	Voxel mask	Multi-organ versatility
LiTS	2023	CT	131 scans (liver+tumor)	Voxel mask	Liver tumor segmentation
KiTS19/21	2019/21	CT	300/489 scans	Voxel mask	Kidney tumor
FLARE 2022	2022	CT	2200 unlabeled + 50 labeled	Voxel mask	Low-resource segmentation
BraTS 2021	2021	MRI	2000+ multi-seq cases	Voxel mask	Brain tumor segmentation
ACDC	2018	MRI	100 patients	Voxel mask	Cardiac chamber seg.
TotalSeg	2023	CT	1228 scans, 104 structs	Voxel mask	Universal anatomy
TotalSeg-MRI	2025	MRI	616 MRI + 527 CT, 80 structs	Voxel mask	MRI universal
MIMIC-CXR	2019	CXR	377K images + reports	Image-text	VLP, classification
CheXpert	2019	CXR	224K images + labels	Image labels	Pathology classification
PadChest	2020	CXR	160K images + reports	Image-text	VLP
VinDr-CXR	2022	CXR	18K with bounding boxes	Bounding box	Detection
NIH ChestXray14	2017	CXR	112K images	Image labels	Classification
MSD	2022	Multi	10 segmentation tasks	Voxel mask	Generalization
ISIC	2019	Dermatology	25K images	Mask + class	Skin lesion
HAM10000	2018	Dermatology	10K images	Class + mask	Skin lesion
BUSI	2020	Ultrasound	780 images	Mask	Breast lesion
Quilt-1M	2023	Pathology	1M image-text pairs	Text caption	Pathology VLP
GLaS	2017	Pathology	165 H&E images	Mask	Gland segmentation
Camelyon16/17	2016/17	Pathology	400+/1000+ WSI	Mask + class	Metastasis detection
BiomedParseData	2025	9 modalities	6M image-mask-text	Mask + text	Text-prompted seg.
CT-RATE	2024	Chest CT	50K volumes + reports	Volume + text	3D VLP
LIDC-IDRI	2011	CT	1018 lung nodule scans	Mask + class	Lung nodule
REFUGE2	2022	Retinal fundus	1200 images	Mask + class	Glaucoma assessment

**Table 6:** Study-quality and evidence assessment of the main models reviewed. Columns report peer-review status, approximate training-data scale, whether external or multi-site validation is reported, the qualitative risk that public evaluation benchmarks overlap large or web-scale pretraining corpora (data leakage), open code, and clinical validation status. Overlap risk is a qualitative judgment, not a measured quantity. Very few reviewed models report prospective or multi-site external clinical validation, which is the main gap between reported benchmark performance and clinical readiness.

Model	Peer Review	Train-Data Scale	External/Multi-Site Val.	Leakage Risk	Open Code	Clinical Validation
MedSAM [43]	Peer-rev.	1.5M image-mask pairs, 10 modalities	Held-out internal; limited external	Moderate	Yes	None (prospective)
SAM-Med2D [72]	Preprint	4.6M images, 19.7M masks	Internal; limited external	Moderate	Yes	None
CLIP-Driven UM [19]	Peer-rev.	14 partial-label CT datasets	BTCV/LiTS/KiTS splits; some cross-dataset	Moderate	Yes	None
BiomedParse [21]	Peer-rev.	6M triples, 45 datasets, 9 modalities	Held-out; presence discriminator; limited external sites	High	Yes	None
BiomedParse-V [22]	Peer-rev. (wksp)	Volumetric extension of above	CVPR 2025 challenge data	High	Partial	None
SAT [92]	Peer-rev.	70+ CT/MRI/PET datasets	Cross-dataset radiology (public)	High	Yes	None
LISA [23]	Peer-rev.	Reasoning-seg + refCOCO (natural)	Natural-image benchmarks	N/A (general)	Yes	None
MedPLIB [103]	Peer-rev.	MeCoVQA region-text dataset	Internal	Moderate	Yes	None
MedCLIP-SAM [113]	Peer-rev.	Reuses MedCLIP + SAM (no retrain)	Multiple public sets	Moderate	Yes	None
SaLIP [114]	Peer-rev. (wksp)	Training-free (zero-shot)	Multiple public sets	Low	Yes	None
SegVol [79]	Peer-rev.	96K unlabeled + 6K labeled CT vol.	Internal held-out	Moderate	Yes	None
MedSAM2 [77]	Preprint	Multi-modal image + video	Internal	Moderate	Yes	None
TotalSegmentator [39]	Peer-rev.	1204 CT, 104 structures	Reproduced across sites; adopted in workflows	Low	Yes	Partial (research/some clinical)
UniverSeg [125]	Peer-rev.	MegaMedical, 16 modalities (few-shot)	Cross-dataset few-shot	Moderate	Yes	None
CT-CLIP/Merlin [59]	Peer-rev.	50K CT volumes + reports (CT-RATE)	Held-out CT-RATE; zero-shot detection	High	Partial	None

## Data Availability

Data sharing is not applicable to this article, as no new datasets were generated or analyzed during the current study. All works discussed are available in the cited references.
